# The impact of vaccination on gender equity: conceptual framework and human papillomavirus (HPV) vaccine case study

**DOI:** 10.1186/s12939-019-1090-3

**Published:** 2020-01-14

**Authors:** Allison Portnoy, Samantha Clark, Sachiko Ozawa, Mark Jit

**Affiliations:** 1 Department of Global Health and Population, Harvard T.H. Chan School of Public Health, 665 Huntington Avenue, Boston, MA 02115 USA; 2 Comparative Health Outcomes, Policy, and Economics (CHOICE) Institute, School of Pharmacy, University of Washington, Seattle, WA USA; 3 Division of Practice Advancement and Clinical Education, UNC Eshelman School of Pharmacy, University of North Carolina, Chapel Hill, NC USA; 4 Department of Maternal and Child Health, UNC Gillings School of Global Public Health, University of North Carolina, Chapel Hill, NC USA; 5 Department of Infectious Disease Epidemiology, London School of Hygiene and Tropical Medicine, London, UK; 6 Modelling and Economics Unit, Public Health England, London, UK

**Keywords:** Human papillomavirus, Vaccination, Gender equity

## Abstract

**Background:**

Although the beneficial effects of vaccines on equity by socioeconomic status and geography are increasingly well-documented, little has been done to extend these analyses to examine the linkage between vaccination and gender equity. In this paper, evidence from the published literature is used to develop a conceptual framework demonstrating the potential impact of vaccination on measures of gender equity. This framework is then applied to human papillomavirus (HPV) vaccination in three countries with different economic and disease burden profiles to establish a proof of concept in a variety of contexts.

**Methods:**

We conducted a literature review examining evidence on the linkage between health outcomes and dimensions of gender equity. We utilized the Papillomavirus Rapid Interface for Modelling and Economics (PRIME) model to estimate cervical cancer incidence and deaths due to HPV types 16/18 by age in each country. We estimated labor force participation and fertility effects from improvements in health, and converted these into inputs consistent with those used to calculate the United Nations Gender Inequality Index to assess gender equity.

**Results:**

In our case study, we found that HPV vaccination among girls could help narrow socioeconomic gender disparities by quantifying the main pathways by which HPV vaccination improves health, which enables improvement in gender equity indicators such as labor force participation and maternal mortality ratios. While these improvements are small when averaged over the entire population, the components measured – labor force participation and maternal mortality ratio – account for 50% of the index scores.

**Conclusions:**

This proof of concept model is a starting point to inform future health and economic analyses that might incorporate the impact of gender equity as an additional impact of vaccination in improving the health and well-being of the population.

## Background

Vaccines are one of the most effective public health innovations in the past century. Every year immunizations save between two and three million lives and avert billions of dollars in costs of illness [1]. Not only are most vaccines cost-effective [2], but childhood immunizations are estimated to have a return on investment of 16 to 44 times the costs in the current decade [3]. Beyond saving lives, improving health and enhancing economic growth, vaccines also deliver wider societal benefits [4]. For example, vaccination can impact children’s cognitive development [5–7] and educational attainment [8–10]. Moreover, vaccination has been found to improve equity by reducing income-related disparities and reducing poverty [11–13]. Although the beneficial effects of vaccines on equity by socioeconomic status and geography are increasingly well-documented, little has been done to extend these analyses to examine the linkage between vaccination and gender equity.

Achieving gender equity and empowering all women and girls is one of the United Nations’ Sustainable Development Goals and a key foundation for a peaceful, prosperous, and sustainable world [14]. Gender inequity relates to health as gender-related power imbalances contribute to excess female mortality, gender-based violence, and limited health-seeking behaviors including lower access to reproductive healthcare [15, 16]. When women and girls receive healthcare, are educated, are not married as a child, and can earn and control income, economies expand and cycles of poverty are broken [17]. While one suggested approach has been to target these conditions directly with policy [17], we sought to examine the link in the other direction from health to development, i.e., whether vaccination in turn can contribute to gender equity.

The linkage between vaccines and gender equity is particularly relevant in the case of the human papillomavirus (HPV) vaccine. HPV vaccine studies demonstrate an almost 100% protection against the previously high-risk HPV strains that cause cervical cancer (types 16 and 18), and HPV vaccines have been shown to be cost-effective in most settings [18–20]. While HPV causes disease in both females and males, globally almost 90% of the HPV vaccine-preventable disease burden falls on females [21]. In women, cervical cancer is the second leading cause of new cases of cancer and cancer death worldwide [22]. Hence we hypothesize that HPV vaccination among girls could help narrow socioeconomic gender disparities by reducing the cervical cancer burden that women experience and increasing the opportunities afforded by improved health.

In this paper, evidence from the published literature is used to develop a conceptual framework demonstrating the potential impact of vaccination on measures of gender equity. This framework is then applied to the HPV vaccine example in three countries with different economic and disease burden profiles to establish the impact of improved health on gender equity in a variety of development contexts. Our objective is to provide a proof of concept model as a starting point to inform future analyses that seek to estimate the health and economic impact of gender equity.

## Methods

### Literature review on the linkage between health outcomes and gender equity dimensions

We first conducted a literature review using two databases (PubMed and Web of Science) examining studies on gender equity and health impact published between January 1, 1980 and December 31, 2018. The search strategy used a combination of free text terms across all search fields. Key search terms included those related to gender equity and health impact (“gender equity” OR “gender inequality” OR “gender gap”) AND “impact” AND “health). Record titles and abstracts were screened by a single reviewer to identify studies that met the inclusion criteria. Articles that did not discuss gender equity, did not look at health impact, were not human studies, contained outcomes from a study already reported in another article, or contained a non-English abstract were excluded. Key working papers regarding the topic of gender equity were also included. From each of the included studies, we extracted information on the dimension(s) of gender equity included and the linkage(s) between health outcomes and gender equity.

### Modeling the impact of HPV vaccination on gender equity

We modeled the impact that HPV vaccination among girls could have on gender equity in a low-, middle-, and high-income country (Tanzania, India, and the United Kingdom), where each country represents a different level of disease burden. Compared to the worldwide annual cervical cancer incidence of 14.0 per 100,000 population [23], Tanzania has a high burden with an incidence of 30.6 [24], India has a medium burden with an incidence of 20.2 [25], and the United Kingdom has a low burden with an incidence of 9.5 per 100,000 population [26]. We utilized the Papillomavirus Rapid Interface for Modelling and Economics (PRIME) model to estimate cervical cancer incidence and deaths due to HPV types 16/18 by age in each country. PRIME is a static, proportional outcomes model that can project the epidemiological and economic outcomes of HPV vaccination of young adolescent women. Specifically, PRIME estimates reductions in age-specific cervical cancer in direct proportion to vaccine efficacy against HPV 16/18, vaccine coverage, and HPV type distribution. It has been used to inform investment decisions by Gavi, the Vaccine Alliance, and the Bill & Melinda Gates Foundation. It has been extensively documented and validated [18]; an Excel-based version of PRIME is freely available at primetool.org. Relevant country- and age-specific inputs for PRIME are provided in the Additional file 1.

In this analysis, we assumed a scenario of 80% coverage of HPV vaccination with a two-dose regimen. The impact of this HPV vaccination scenario on cervical cancer incidence and death projected by PRIME is augmented with the extracted literature review data on the linkages between health outcomes and dimensions of gender equity. Based on evidence from the literature, we developed a conceptual pathway by which HPV vaccination can improve healthy life expectancy in women, which can in turn improve their labor force participation and decrease fertility, and hence narrow a key source of gender disparities in many countries [27]. Labor force participation and maternal mortality were selected as criteria for reducing gender disparities given their inclusion in multiple gender equity measures and linkage to improved female health [15, 27–35]. We were unable to quantify the effect of improved healthy life expectancy on maternal mortality directly, but instead assumed that decreased fertility led to an associated decrease in maternal mortality.

To quantify the magnitude of these relationships, we first converted years of life expectancy to years of healthy life expectancy utilizing life tables and healthy life expectancy at birth and at age 60 from the World Health Organization (WHO) for Tanzania, India, and the United Kingdom [36]. Given currently published WHO estimates of healthy life expectancy at birth and at age 60, we created multipliers from the level of life expectancy to healthy life expectancy at these two ages. We used these multipliers to adjust the life expectancy curves from birth to age 60 to approximate years of healthy life expectancy for each age. Between age 60 and 100, we used the same approach given the points of healthy life expectancy at age 60 from the WHO and an assumption of 0.5 for remaining healthy life expectancy at age 100 to create new multipliers to adjust the life expectancy curves.

Using these estimates of healthy life expectancy, we assumed that the decreased cervical cancer incidence and deaths from HPV vaccination yielded decreased years lived with disability (YLDs) and decreased years of life lost (YLLs). The ratio of the decreased YLDs and YLLs to healthy life years gained compared to total YLDs and YLLs to total healthy life expectancy was interpreted as the percentage improvement in health due to HPV vaccination. This percentage improvement in health translated to percentage increases in labor force participation and percentage reductions in fertility rate, utilizing percentages calculated from evidence identified through the literature review. According to a working paper by Bloom, et al., we assumed that a 5% improvement in health from HPV vaccination translated to a 5.9% increase in female labor force participation and a 2.4% reduction in fertility [27]. These percentages reflect years of healthy life lost due to disability for the age groups 15–29 and 30–49, which are assumed to span the potential period of fertility (ages 15–35) and child-rearing (ages 35–50) for women. We also examined what the improvements would be if the magnitude of the associations were half or twice these values through sensitivity analyses.

The impact of HPV vaccination on overall gender disparities was assessed by converting previously estimated labor force participation and fertility effects into inputs consistent with those used to calculate the United Nations (UN) Gender Inequality Index (GII) [37, 38]. The GII is based on the UN Human Development Index and provides a quantitative measure of the human development costs of gender inequality along three dimensions: (1) reproductive health (e.g., maternal mortality ratio and adolescent birth rates); (2) empowerment (e.g., proportion of parliamentary seats held and secondary education attainment); and (3) economic status (e.g., labor force participation). While labor force participation could be directly incorporated into the GII calculation, fertility changes had to be converted to a corresponding GII input (in this case, maternal mortality ratios) using estimates of the relationship between fertility and maternal mortality from the published literature [39]. We therefore estimated the years of employment gained according to the percentage improvements in labor force participation and associated improvements in healthy life expectancy when applied across the total population by five-year age groups. We multiplied the years of employment gained by country gross domestic product (GDP) per capita in order to estimate improvements in economic productivity. We assumed that all additional years of healthy life expectancy during productive working age (i.e., ages 15 to 49) translated to years of employment gained. Similarly, we estimated the maternal deaths averted according to the percentage improvements in maternal mortality ratios from associated reductions in fertility rates.

Once the relevant inputs were derived, the impact of HPV vaccination on GII scores were calculated by subtracting the current index value from the counterfactual index value under a hypothetical 0% HPV vaccine coverage scenario. Since higher GII scores are associated with greater inequality, the difference between these two values can be interpreted as the reduction in inequality associated with HPV vaccination.

## Results

### Findings from the literature review on the linkage between health outcomes and gender equity

Our review of dimensions of gender equity identified five relevant articles [15, 33, 34, 37, 38] on gender equity indices. The gender development index (GDI) of the United Nations Development Program (UNDP) includes: life expectancy, literacy rate, school enrollment, and economic activity [15, 37]. The gender empowerment measures (GEM) of UNDP includes: female representation in the government and the labor force [15]. The Gender Equity Index (GEI) includes: education, economic activity, and empowerment (i.e., political participation, representation in government positions, law-making) [37]. The Gender Gap Index (GGI) includes: economic development, education, health, survival, and political participation [37]. The Gender Parity Index (GPI) includes: education, economic activity, and parliamentary representation [33]. The UN GII includes: reproductive health (i.e., maternal mortality and adolescent fertility), empowerment (i.e., parliamentary representation and secondary education attainment) and labor force participation [37, 38]. The Labour Force Gender Index (LFGI) includes: child-care responsibility, occupation segregation, labor force participation, and level of education [34].

**Fig. 1:**
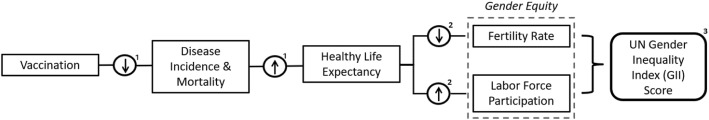
Conceptual flow diagram. *Note:* [1] Feikin et al., 2016 [50]. [2] Bloom et al., 2016 [27]. [3] United Nations Development Programme, 2018 [35]

The factors included in these gender equity indices contributed to our second literature review examining linkages between gender equity and health. Our literature search resulted in 205 articles on PubMed and 179 articles on Web of Science, of which 12 met inclusion criteria [27, 38, 40–49]. These articles provided us with a list of relevant indicators that are linked to gender equity and health (Appendix C in Additional file 1). In order to link health gains from vaccination to improvements in gender equity, we examined this list for indicators that could be quantitatively linked to improvements in health from the literature. Bloom, et al. [27], provided this quantitative linkage with an estimated relationship between percentage improvement in health and percentage improvements in both labor force participation and fertility rate. Relying on this study in combination with the relationship between vaccination and health [50] as well as the UN GII [35], we developed a flow diagram for the conceptual framework linking vaccination to gender equity, shown in Fig. 1.

### Calculating healthy life expectancy

**Fig. 2:**
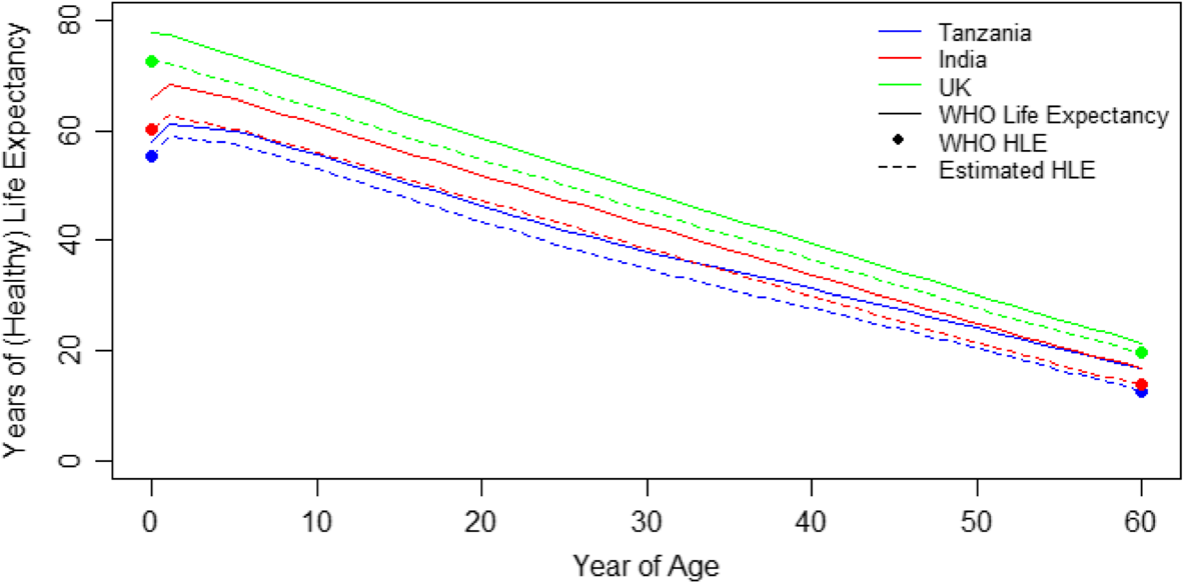
Estimated years of life expectancy and healthy life expectancy (HLE) for Tanzania, India, and the United Kingdom. *Note: The top curve for each country represents World Health Organization (WHO) life expectancy estimates, which are adjusted down to our estimated healthy life expectancy estimates in the bottom curve, anchoring on the HLE at birth and HLE at age 60 estimates from WHO [36]

The results of our healthy life expectancy calculations are shown in Fig. 2. The solid lines show the WHO life expectancy curves and the points show WHO estimates of healthy life expectancy at birth and at age 60 for Tanzania, India, and the United Kingdom. The dotted lines show our calculations of healthy life expectancy by each year of age, anchoring on the previous curves and healthy life expectancy estimates.

### Impact of HPV vaccination

**Fig. 3:**
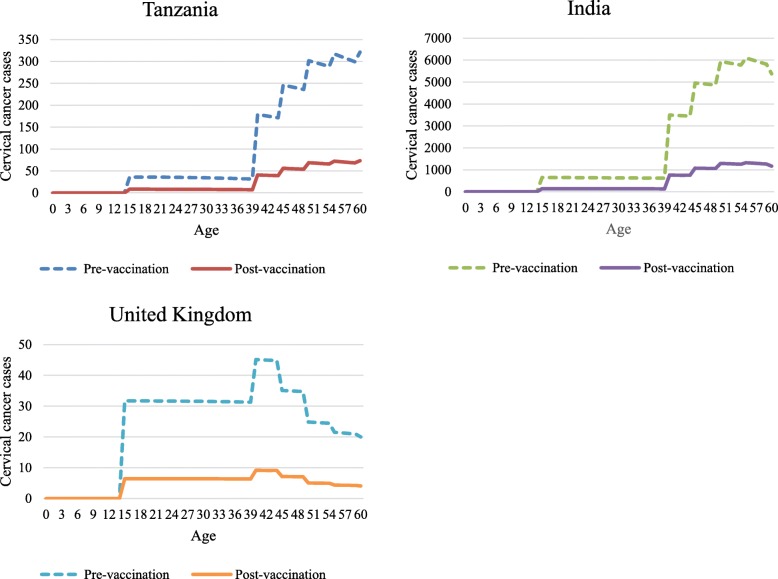
Impact of HPV vaccination on cervical cancer cases by age in a cohort of women vaccinated at 9 years old in Tanzania, India, and the United Kingdom

Overall, HPV vaccination is expected to decrease cervical cancer cases and deaths by approximately 80% in Tanzania, India, and the United Kingdom based on PRIME projections. For women of reproductive age (15 to 49 years), the median percentage improvement in healthy life expectancy due to reductions in cervical cancer cases and deaths is 51% in Tanzania, 52% in India, and 49% in the United Kingdom. The impact that HPV vaccination is projected to have in Tanzania, India, and the United Kingdom is shown in Fig. 3.

### Impact of HPV vaccination on gender equity dimensions

Assuming a 5% improvement in health from HPV vaccination translates to a 5.9% increase in female labor force participation [27], years of employment gained among women who averted cervical cancer by five-year age of onset ranged from 165 to 920 years in Tanzania, 1404–9600 years in India, and 38–78 years in the United Kingdom for women aged 15 to 49 years (Table 1). The same 5% improvement in health and resulting fertility reduction of 2.4% was estimated to lead to a corresponding decrease in the maternal mortality ratio (per 100,000 live births) of 4.5%. This decline in maternal mortality was associated with 1.4 fewer maternal deaths in Tanzania, 2.3 fewer maternal deaths in India, and approximately no change in maternal deaths in the United Kingdom.

**Table 1:** Years of life saved and years of employment gained and maternal deaths averted among women with averted cervical cancer in Tanzania, India, and the United Kingdom

Age Group	Tanzania			India			United Kingdom		
	Years of life saved	Years of employment gained	Maternal deaths averted	Years of life saved	Years of employment gained	Maternal deaths averted	Years of life saved	Years of employment gained	Maternal deaths averted
15–19	2672	313	0.03	45,643	2492	0.07	725	65	< 0.01
20–24	2386	278	0.03	41,474	2216	0.07	668	58	< 0.01
25–29	2107	242	0.04	37,345	1941	0.07	612	51	< 0.01
30–34	1836	203	0.05	33,259	1672	0.08	556	45	< 0.01
35–39	1578	165	0.05	29,218	1404	0.09	500	38	< 0.01
40–44	8510	819	0.43	176,959	7987	0.69	1121	78	< 0.01
45–49	10,652	920	0.80	234,004	9600	1.22	1076	66	< 0.01
Total	29,742	2939	1.43	597,902	27,312	2.29	5258	401	< 0.01

The years of employment gained from improvements in health due to reduced cervical cancer incidence also contributes to increased economic output. The improvements in economic productivity from years of employment gained by female workers could be approximately $4.7 million in Tanzania, $24 million in India, and $18 million in the United Kingdom (in US$2015).

In our sensitivity analysis examining the lower range with half the magnitude of this association, which assumes that a 5% improvement in health from HPV vaccination translate to a 2.95% increase in female labor force participation, years of employment gained among women who averted cervical cancer ranged from 82 to 460 years in Tanzania, 702–4800 years in India, and 19–39 years in the United Kingdom for women aged 15 to 49 years. In terms of economic productivity, this translated to approximately $2.4 million in Tanzania, $12 million in India, and $8.9 million in the United Kingdom. Assuming a 5% improvement in health from HPV vaccination translated to a 1.2% decrease in fertility rate, the associated impact on maternal mortality was 0.72 maternal deaths averted in Tanzania, 2.15 maternal deaths averted in India, and approximately no change in maternal deaths in the United Kingdom.

In our sensitivity analysis examining the upper range with double the magnitude of association, which assumed that a 5% improvement in health from HPV vaccination translate to a 11.8% increase in female labor force participation, years of employment gained among women who averted cervical cancer ranged from 330 to 1840 years in Tanzania, 2808–19,200 years in India, and 76–156 years in the United Kingdom for women aged 15 to 49 years. In terms of economic productivity, this translated to approximately $9.4 million in Tanzania, $48 million in India, and $36 million in the United Kingdom. Assuming a 5% improvement in health from HPV vaccination translated to a 4.8% decrease in fertility rate, the associated impact on maternal mortality was 2.9 maternal deaths averted in Tanzania, 8.6 maternal deaths averted in India, and approximately no change in maternal deaths in the United Kingdom.

Comparing GII scores calculated based on current HPV vaccination levels and under the counterfactual scenario of 0% HPV vaccination, improvements in labor force participation and maternal survival were both associated with a decrease in the level of gender inequality (i.e., increase in the level of gender equity) in the three countries of interest. Compared to the status quo GII scores in Tanzania, India, and the United Kingdom (0.537, 0.524, and 0.116, respectively), the absolute changes in GII scores were small (0.0000021 (0.0000019–0.000013), 0.0000017 (0.0000017–0.0000032), and 0.0000002 (0.0000002–0.0000004), respectively) because cervical cancer is a rare outcome and GII index scores are estimated on a 0–1 scale. While the index comprises multiple dimensions, labor force participation and maternal mortality ratio account for approximately 50% of the index score.

## Discussion

We developed a conceptual framework that links vaccination and gender equity utilizing existing literature. In our case study, we found that HPV vaccination could help narrow socioeconomic gender disparities by quantifying the main pathways by which HPV vaccination improves health, which enables improvement in gender equity indicators such as labor force participation and maternal mortality ratios. Specifically, we found that the years of employment gained among women with averted cervical cancer ranged from 165 to 920 years in Tanzania, 1404–9600 years in India, and 38–78 years in the United Kingdom for women aged 15 to 49 years. The averted maternal deaths among women with averted cervical cancer was 1.4 in Tanzania and 2.3 in India. These improvements likewise translate to improvements in gender equity. While these improvements are small when averaged over the entire population, the components measured – labor force participation and maternal mortality ratio – account for 50% of the index score.

While the magnitude of improvement we found is small due to the rarity of cervical cancer, we have likely underestimated the benefits. This is because our model did not include many other benefits of HPV vaccination such as indirect (herd) effects, social stigma associated with cervical and other HPV-related cancers, reductions in time-consuming and anxiety-inducing cervical screening and treatment rounds, as well as effects on non-cervical HPV-related disease such as vulvar, vaginal, anal and oropharyngeal cancers [21, 51]. We also only assumed vaccination coverage of girls, per the WHO recommendations for routine immunization [52], whereas many countries also vaccinate boys. However, even if countries were to vaccinate both girls and boys, we would still likely see a gender equity benefit due to the health benefits of HPV vaccination primarily accruing among girls [53]. Furthermore, we have only explored two of the many pathways between improved health and gender equity (via female labor participation and maternal mortality) because of the availability of existing models of these pathways and their inclusion in the GII. In particular, the pathway between improved health and gender equity via improvements in female educational attainment is relevant [5, 8, 9, 38, 44, 46, 54], but excluded in this analysis due to limited data and the late age of onset of cervical cancer. Additionally, given the late age of onset of HPV compared to other vaccine-preventable diseases, it may be possible that the impact of vaccination against childhood diseases could have a larger impact on gender equity. Despite these limitations, our work provides for the first time, to our knowledge, both a proof of concept and quantitative links between vaccination and gender equity.

In this analysis, we selected HPV vaccination as a case study for gender equity given that its health benefits are predominantly in women [53]. If we examined a vaccine that was more gender-neutral in the acquisition of health benefits such as measles or rotavirus, health improvements among both men and women would impact both labor force participation and fertility rates. According to Bloom, et al., male health improvements increase the fertility rate, which would likewise decrease the female labor force participation rate [27]. Therefore, if a health intervention such as vaccination improved the health of both sexes equally, the macroeconomic effects may be counteracting. However, given that an economy has transitioned to a regime of “declining fertility and increasing educational investments,” the dynamic equilibrium model developed by Bloom, et al., found that gender-neutral health improvements still improved overall economic growth via female labor force participation and declining fertility [27]. The paper further states that health improvements among both sexes also supports the transition from a high fertility regime to a low fertility regime. In the case of vaccination in LMICs, the net improvement of a gender-neutral vaccination might therefore lead to improvements in gender equity, although likely to a lesser extent than an HPV vaccination program. Hence, we can conclude that most immunization programs are likely to contribute to gender equity as long as they achieve equal uptake among males and females.

Equity is a key criterion for health technology assessments in many countries [55, 56] and by organizations such as the Bill & Melinda Gates Foundation [57]. Up to now, most of the literature around vaccines and equity has focused on socioeconomic [58–60] and geographical [61–63] dimensions of equity. Additionally, suggestions to address gender equity and female empowerment as part of the Sustainable Development Goals have focused on policies around dimensions of gender equity, rather than health [17]. Our work shows that vaccines may also help to narrow gender disparities by a conceptual framework that links improvements in health to dimensions of gender equity, and that the magnitude of such effects can be quantified. Furthermore, we have shown that those effects improve the same dimensions that have been identified as essential to gender equity, where gaps still remain [17, 64].

Given the importance of gender equity to development, this suggests that decision-making bodies such as national immunization technical advisory groups (NITAGs) and donors may wish to consider vaccine impact on gender equity as one of the decision-making criteria around new vaccine introductions. Future research should be conducted to examine these gender equity benefits beyond this case study of three countries from the lens of HPV vaccination.

This analysis presents a number of limitations. First, our conceptual framework and quantitative associations rely on the available published literature, which remains limited on this topic. Specifically, we rely heavily on Bloom, et al. [27], which reports on a dynamic general equilibrium model of economic development across countries, and may not be generalizable to the settings that we feature in this analysis. Additionally, as the percentages obtained from this model are applied across all ages equally, the results of our analysis would be affected if the age distribution of disease by country is heavily skewed towards younger or older women. In order to address this limitation, we conducted a sensitivity analysis on the impact of improvements in health on female labor force participation and fertility with lower and upper bounds of 0.5 and 2 times multipliers, which would encompass environments where the age distribution of disease trends toward older women, i.e., lower reductions in fertility and improvements in labor force participation, or younger women, i.e., greater reductions in fertility and improvements in labor force participation. Second, in order to preserve the decreased life expectancy at birth and the increased life expectancy for individuals who survive infancy in our calculations of healthy life expectancy, we applied a constant to life expectancy between birth and age 60, but did not apply the constant beyond age 60. While this approximates the trend of the life expectancy curve, this approach results in a discontinuity at age 60 that should be smooth, but is necessary to provide a reasonable estimate of healthy life expectancy between age 60 and 100. Third, we assumed that the relationship between health and equity is the same across countries, but it may vary, as it contains inherent cultural and infrastructural assumptions that are not country-invariant. Fully addressing the relationship between health and equity in each country would require econometric analysis at the country level. We were also unable to control for changes in female labor force participation due to events unrelated to vaccination. Fourth, the individual-level impact of reduced cervical cancer on female labor force participation and fertility are less certain compared to vaccines which cause sequelae from early childhood. We might still expect to see individual-level fertility effects with HPV vaccine due to health expectations, e.g., if women are less likely to expect to be ill and have fertility affected in later life due to cervical cancer, then they may be more inclined to invest in education, delay childbirth, or even reduce fertility. Additionally, a woman who has cervical cancer and recovers may well have her fertility negatively affected since many of the treatments for cervical cancer affect fertility [65]. In order to address this uncertainty, we have conducted a sensitivity analysis on the fertility effect. Finally, the model presented here does not represent a causal relationship between HPV vaccination and gender equity.

While our analysis focused on reductions in gender inequity from overall improvements to healthy life expectancy, Finlay and Lee (2018) have examined the causal linkages between improvements in reproductive health, specifically, and the economic empowerment dimensions of gender equity [66]. This analysis relied on empirical evidence to draw similar conclusions that reproductive health improvements can and do empower women through effects on education, labor force participation, and childbearing. However, our analysis takes these results a step further by providing a proof of concept model for how a public health intervention such as vaccination could have a quantifiable impact on gender equity. Finally, there remains uncertainty in improvements in health, labor force participation, and fertility rate, particularly as applied to these specific country contexts. However, we hope to have addressed part of this uncertainty by including ranges from sensitivity analyses.

## Conclusions

Gender equity is one of the United Nations’ Sustainable Development Goals and a key dimension of inclusive economic growth. It has been associated with improvements in health outcomes for women and children, in addition to economic growth. This proof of concept model is a starting point to inform future health and economic analyses that might incorporate the impact of gender equity as an additional impact of vaccination in improving the health and well-being of the population. Reduced HPV incidence may lead to improved gender equity through improvements in female contributions to the labor market and economic productivity, improvements in female education, lower fertility, and reduced youth dependency.

## Supplementary information

**Additional file 1:** Appendix A. Papillomavirus Rapid Interface for Modelling and Economics (PRIME) tool country-level inputs for India, Tanzania, and the United Kingdom. Appendix B. Cervical cancer incidence and mortality and all-cause mortality by age for India, Tanzania, and the United Kingdom. Appendix C. List of linkages between gender equity and health.

## Data Availability

The datasets generated during and/or analyzed during the current study are available from the corresponding author on reasonable request.

## Abbreviations

GDI: Gender development index
GDP: Gross domestic product
GEI: Gender equity index
GEM: Gender empowerment measures
GGI: Gender gap index
GII: Gender inequality index
GPI: Gender parity index
HPV: Human papillomavirus
LFGI: Labour force gender index
NITAG: National Immunization Technical Advisory Group
PRIME: Papillomavirus Rapid Interface for Modelling and Economics
UN: United Nations
UNDP: United Nations Development Program
WHO: World Health Organization
YLD: Year(s) lived with disability
YLL: Year(s) of life lost
