# Supplementary material for: The impact of vaccination on gender equity: conceptual framework and human papillomavirus (HPV) vaccine case study
Source: Int J Equity Health. 2020 Jan 14;19:10. doi: 10.1186/s12939-019-1090-3 (PMC6961353; doi:10.1186/s12939-019-1090-3)
Supplement: Supplementary file 1 — Additional file 1: Appendix A. Papillomavirus Rapid Interface for Modelling and Economics (PRIME) tool country-level inputs for India, Tanzania, and the United Kingdom. Appendix B. Cervical cancer incidence and mortality and all-cause mortality by age for India, Tanzania, and the United Kingdom. Appendix C. List of linkages between gender equity and health. [file 12939_2019_1090_MOESM1_ESM.pdf]

Appendix A. Papillomavirus Rapid Interface for Modelling and Economics (PRIME) tool country-level inputs for India, Tanzania, and the United Kingdom.

Country	India	Tanzania	United Kingdom
Birth cohort size (female)	11,719,276	395,953	395,953
Cohort size at vaccination age (female)	11,973,174	338,891	338,891
Coverage (all doses)	80%	80%	80%
Vaccine efficacy vs HPV 16/18	100%	100%	100%
Target age group	12	12	12
DALYs for cancer diagnosis	0.08	0.08	0.08
DALYs for non-terminal cancer sequelae (per year)	0.17	0.17	0.04
DALYs for terminal cancer	0.78	0.78	0.78
Discount rate	3.0%	3.0%	3.0%
Proportion of cervical cancer cases that are due to HPV 16/18	81.9%	74.3%	76.6%

Appendix B. Cervical cancer incidence and mortality and all-cause mortality by age for India, Tanzania, and the United Kingdom.

Age	India			Tanzania			United Kingdom		
	Cervical cancer incidence	Cervical cancer mortality	All-cause mortality	Cervical cancer incidence	Cervical cancer mortality	All-cause mortality	Cervical cancer incidence	Cervical cancer mortality	All-cause mortality
**0**	0	0	0.0527	0	0	0.06974	0	0	0.00979125
**1**	0	0	0.00511	0	0	0.01167	0	0	0.0003565
**2**	0	0	0.00511	0	0	0.01167	0	0	0.0003565
**3**	0	0	0.00511	0	0	0.01167	0	0	0.0003565
**4**	0	0	0.00511	0	0	0.01167	0	0	0.0003565
**5**	0	0	0.00143	0	0	0.00196	0	0	0.000179
**6**	0	0	0.00143	0	0	0.00196	0	0	0.000179
**7**	0	0	0.00143	0	0	0.00196	0	0	0.000179
**8**	0	0	0.00143	0	0	0.00196	0	0	0.000179
**9**	0	0	0.00143	0	0	0.00196	0	0	0.000179
**10**	0	0	0.00102	0	0	0.00139	0	0	0.00018075
**11**	0	0	0.00102	0	0	0.00139	0	0	0.00018075
**12**	0	0	0.00102	0	0	0.00139	0	0	0.00018075
**13**	0	0	0.00102	0	0	0.00139	0	0	0.00018075
**14**	0	0	0.00102	0	0	0.00139	0	0	0.00018075
**15**	0.000074	0.000025	0.00158	0.00014	0.000054	0.00116	0.000106	0.00001	0.00030775
**16**	0.000074	0.000025	0.00158	0.00014	0.000054	0.00116	0.000106	0.00001	0.00030775
**17**	0.000074	0.000025	0.00158	0.00014	0.000054	0.00116	0.000106	0.00001	0.00030775
**18**	0.000074	0.000025	0.00158	0.00014	0.000054	0.00116	0.000106	0.00001	0.00030775
**19**	0.000074	0.000025	0.00158	0.00014	0.000054	0.00116	0.000106	0.00001	0.00030775
**20**	0.000074	0.000025	0.00212	0.00014	0.000054	0.00292	0.000106	0.00001	0.00042025
**21**	0.000074	0.000025	0.00212	0.00014	0.000054	0.00292	0.000106	0.00001	0.00042025
**22**	0.000074	0.000025	0.00212	0.00014	0.000054	0.00292	0.000106	0.00001	0.00042025
**23**	0.000074	0.000025	0.00212	0.00014	0.000054	0.00292	0.000106	0.00001	0.00042025
**24**	0.000074	0.000025	0.00212	0.00014	0.000054	0.00292	0.000106	0.00001	0.00042025
**25**	0.000074	0.000025	0.00198	0.00014	0.000054	0.00548	0.000106	0.00001	0.00052675
**26**	0.000074	0.000025	0.00198	0.00014	0.000054	0.00548	0.000106	0.00001	0.00052675
**27**	0.000074	0.000025	0.00198	0.00014	0.000054	0.00548	0.000106	0.00001	0.00052675
**28**	0.000074	0.000025	0.00198	0.00014	0.000054	0.00548	0.000106	0.00001	0.00052675
**29**	0.000074	0.000025	0.00198	0.00014	0.000054	0.00548	0.000106	0.00001	0.00052675
**30**	0.000074	0.000025	0.00224	0.00014	0.000054	0.00837	0.000106	0.00001	0.0007385
**31**	0.000074	0.000025	0.00224	0.00014	0.000054	0.00837	0.000106	0.00001	0.0007385

Age	India			Tanzania			United Kingdom		
	Cervical cancer incidence	Cervical cancer mortality	All-cause mortality	Cervical cancer incidence	Cervical cancer mortality	All-cause mortality	Cervical cancer incidence	Cervical cancer mortality	All-cause mortality
**32**	0.000074	0.000025	0.00224	0.00014	0.000054	0.00837	0.000106	0.00001	0.0007385
**33**	0.000074	0.000025	0.00224	0.00014	0.000054	0.00837	0.000106	0.00001	0.0007385
**34**	0.000074	0.000025	0.00224	0.00014	0.000054	0.00837	0.000106	0.00001	0.0007385
**35**	0.000074	0.000025	0.00264	0.00014	0.000054	0.01052	0.000106	0.00001	0.001069
**36**	0.000074	0.000025	0.00264	0.00014	0.000054	0.01052	0.000106	0.00001	0.001069
**37**	0.000074	0.000025	0.00264	0.00014	0.000054	0.01052	0.000106	0.00001	0.001069
**38**	0.000074	0.000025	0.00264	0.00014	0.000054	0.01052	0.000106	0.00001	0.001069
**39**	0.000074	0.000025	0.00264	0.00014	0.000054	0.01052	0.000106	0.00001	0.001069
**40**	0.000419	0.000178	0.00337	0.000803	0.000347	0.01058	0.000153	0.000026	0.001622
**41**	0.000419	0.000178	0.00337	0.000803	0.000347	0.01058	0.000153	0.000026	0.001622
**42**	0.000419	0.000178	0.00337	0.000803	0.000347	0.01058	0.000153	0.000026	0.001622
**43**	0.000419	0.000178	0.00337	0.000803	0.000347	0.01058	0.000153	0.000026	0.001622
**44**	0.000419	0.000178	0.00337	0.000803	0.000347	0.01058	0.000153	0.000026	0.001622
**45**	0.000604	0.000281	0.00459	0.001165	0.000528	0.01023	0.00012	0.000029	0.0025685
**46**	0.000604	0.000281	0.00459	0.001165	0.000528	0.01023	0.00012	0.000029	0.0025685
**47**	0.000604	0.000281	0.00459	0.001165	0.000528	0.01023	0.00012	0.000029	0.0025685
**48**	0.000604	0.000281	0.00459	0.001165	0.000528	0.01023	0.00012	0.000029	0.0025685
**49**	0.000604	0.000281	0.00459	0.001165	0.000528	0.01023	0.00012	0.000029	0.0025685
**50**	0.00074	0.000361	0.00674	0.001504	0.000736	0.01103	0.000086	0.000036	0.004117
**51**	0.00074	0.000361	0.00674	0.001504	0.000736	0.01103	0.000086	0.000036	0.004117
**52**	0.00074	0.000361	0.00674	0.001504	0.000736	0.01103	0.000086	0.000036	0.004117
**53**	0.00074	0.000361	0.00674	0.001504	0.000736	0.01103	0.000086	0.000036	0.004117
**54**	0.00074	0.000361	0.00674	0.001504	0.000736	0.01103	0.000086	0.000036	0.004117
**55**	0.000786	0.000407	0.01164	0.001671	0.000952	0.01433	0.000076	0.000043	0.0064475
**56**	0.000786	0.000407	0.01164	0.001671	0.000952	0.01433	0.000076	0.000043	0.0064475
**57**	0.000786	0.000407	0.01164	0.001671	0.000952	0.01433	0.000076	0.000043	0.0064475
**58**	0.000786	0.000407	0.01164	0.001671	0.000952	0.01433	0.000076	0.000043	0.0064475
**59**	0.000786	0.000407	0.01164	0.001671	0.000952	0.01433	0.000076	0.000043	0.0064475
**60**	0.000735	0.00045	0.02103	0.00182	0.001206	0.01951	0.000073	0.00005	0.00973275
**61**	0.000735	0.00045	0.02103	0.00182	0.001206	0.01951	0.000073	0.00005	0.00973275
**62**	0.000735	0.00045	0.02103	0.00182	0.001206	0.01951	0.000073	0.00005	0.00973275
**63**	0.000735	0.00045	0.02103	0.00182	0.001206	0.01951	0.000073	0.00005	0.00973275
**64**	0.000735	0.00045	0.02103	0.00182	0.001206	0.01951	0.000073	0.00005	0.00973275

Age	India			Tanzania			United Kingdom		
	Cervical cancer incidence	Cervical cancer mortality	All-cause mortality	Cervical cancer incidence	Cervical cancer mortality	All-cause mortality	Cervical cancer incidence	Cervical cancer mortality	All-cause mortality
**65**	0.000634	0.000492	0.03234	0.002023	0.001512	0.03168	0.000074	0.000057	0.01595025
**66**	0.000634	0.000492	0.03234	0.002023	0.001512	0.03168	0.000074	0.000057	0.01595025
**67**	0.000634	0.000492	0.03234	0.002023	0.001512	0.03168	0.000074	0.000057	0.01595025
**68**	0.000634	0.000492	0.03234	0.002023	0.001512	0.03168	0.000074	0.000057	0.01595025
**69**	0.000634	0.000492	0.03234	0.002023	0.001512	0.03168	0.000074	0.000057	0.01595025
**70**	0.00047	0.000512	0.05607	0.002239	0.001864	0.0528	0.000077	0.000069	0.0266435
**71**	0.00047	0.000512	0.05607	0.002239	0.001864	0.0528	0.000077	0.000069	0.0266435
**72**	0.00047	0.000512	0.05607	0.002239	0.001864	0.0528	0.000077	0.000069	0.0266435
**73**	0.00047	0.000512	0.05607	0.002239	0.001864	0.0528	0.000077	0.000069	0.0266435
**74**	0.00047	0.000512	0.05607	0.002239	0.001864	0.0528	0.000077	0.000069	0.0266435
**75**	0.000247	0.000513	0.0787	0.002494	0.002267	0.08688	0.000093	0.000104	0.04687725
**76**	0.000247	0.000513	0.0787	0.002494	0.002267	0.08688	0.000093	0.000104	0.04687725
**77**	0.000247	0.000513	0.0787	0.002494	0.002267	0.08688	0.000093	0.000104	0.04687725
**78**	0.000247	0.000513	0.0787	0.002494	0.002267	0.08688	0.000093	0.000104	0.04687725
**79**	0.000247	0.000513	0.0787	0.002494	0.002267	0.08688	0.000093	0.000104	0.04687725
**80**	0.000247	0.000513	0.12099	0.002494	0.002267	0.13693	0.000093	0.000104	0.08301425
**81**	0.000247	0.000513	0.12099	0.002494	0.002267	0.13693	0.000093	0.000104	0.08301425
**82**	0.000247	0.000513	0.12099	0.002494	0.002267	0.13693	0.000093	0.000104	0.08301425
**83**	0.000247	0.000513	0.12099	0.002494	0.002267	0.13693	0.000093	0.000104	0.08301425
**84**	0.000247	0.000513	0.12099	0.002494	0.002267	0.13693	0.000093	0.000104	0.08301425
**85**	0.000247	0.000513	0.18182	0.002494	0.002267	0.20789	0.000093	0.000104	0.14132575
**86**	0.000247	0.000513	0.18182	0.002494	0.002267	0.20789	0.000093	0.000104	0.14132575
**87**	0.000247	0.000513	0.18182	0.002494	0.002267	0.20789	0.000093	0.000104	0.14132575
**88**	0.000247	0.000513	0.18182	0.002494	0.002267	0.20789	0.000093	0.000104	0.14132575
**89**	0.000247	0.000513	0.18182	0.002494	0.002267	0.20789	0.000093	0.000104	0.14132575
**90**	0.000247	0.000513	0.2671	0.002494	0.002267	0.30365	0.000093	0.000104	0.22859225
**91**	0.000247	0.000513	0.2671	0.002494	0.002267	0.30365	0.000093	0.000104	0.22859225
**92**	0.000247	0.000513	0.2671	0.002494	0.002267	0.30365	0.000093	0.000104	0.22859225
**93**	0.000247	0.000513	0.2671	0.002494	0.002267	0.30365	0.000093	0.000104	0.22859225
**94**	0.000247	0.000513	0.2671	0.002494	0.002267	0.30365	0.000093	0.000104	0.22859225
**95**	0.000247	0.000513	0.38355	0.002494	0.002267	0.42633	0.000093	0.000104	0.3492875
**96**	0.000247	0.000513	0.38355	0.002494	0.002267	0.42633	0.000093	0.000104	0.3492875
**97**	0.000247	0.000513	0.38355	0.002494	0.002267	0.42633	0.000093	0.000104	0.3492875

Age	India			Tanzania			United Kingdom		
	Cervical cancer incidence	Cervical cancer mortality	All-cause mortality	Cervical cancer incidence	Cervical cancer mortality	All-cause mortality	Cervical cancer incidence	Cervical cancer mortality	All-cause mortality
**98**	0.000247	0.000513	0.38355	0.002494	0.002267	0.42633	0.000093	0.000104	0.3492875
**99**	0.000247	0.000513	0.38355	0.002494	0.002267	0.42633	0.000093	0.000104	0.3492875
**100**	0.000247	0.000513	1	0.002494	0.002267	1	0.000093	0.000104	1

Appendix C. List of linkages between gender equity and health.

Maternal health →

- Women's human capital (Albanesi & Olivetti, 2014) (40)
- Labor force participation (Albanesi & Olivetti, 2016; Bloom et al., 2009; Bloom et al., 2016) (27, 41, 42)
- Fertility (Albanesi & Olivetti, 2016; Bloom et al., 2009; Bloom et al., 2016) (27, 41, 42)
- Economic productivity (Onarheim et al., 2016) (48)

Life expectancy →

- Educational attainment (Jayachandran & Lleras-Muney, 2009) (45)

Child health and mortality →

- Maternal gender equity (Brinda et al., 2015) (43)
- Educational attainment and/or performance (Gakidou et al., 2010; Jukes, 2005) (44, 46)

Gender equity →

- Healthy life expectancy at birth (Kim & Kim, 2014) (38)
- Health of women and girls (Payne, 2015) (49)

Note: King et al., 2018 was a review article covering multiple linkages (47).
